# (*E*)-4-Hy­droxy-*N*′-(3-hy­droxy-4-meth­oxy­benzyl­idene)benzohydrazide

**DOI:** 10.1107/S1600536811036579

**Published:** 2011-09-14

**Authors:** Hoong-Kun Fun, Jirapa Horkaew, Suchada Chantrapromma

**Affiliations:** a X-ray Crystallography Unit, School of Physics, Universiti Sains Malaysia, 11800 USM, Penang, Malaysia; b Department of Chemistry and Center of Excellence for Innovation in Chemistry, Faculty of Science, Prince of Songkla University, Hat-Yai, Songkhla 90112, Thailand; c Crystal Materials Research Unit, Department of Chemistry, Faculty of Science, Prince of Songkla University, Hat-Yai, Songkhla 90112, Thailand

## Abstract

The mol­ecule of the title benzohydrazide derivative, C_15_H_14_N_2_O_4_, exists in a *trans* conformation with respect to the C=N double bond and is twisted, the dihedral angle between the two benzene rings being 24.17 (6)°. The meth­oxy group is almost co-planar with respect to the attached benzene ring [C_m_—O—C—C (m = meth­yl) = −1.45 (17)°]. In the crystal, the mol­ecules are linked by N—H⋯O and O—H⋯O hydrogen bonds into sheets parallel to the *bc* plane. These sheets are further connected into a three-dimensional network by weak C—H⋯O and C—H⋯π inter­actions.

## Related literature

For bond-length data, see: Allen *et al.* (1987 ▶). For related structures, see: Li & Ban (2009 ▶); Zhang (2011 ▶). For background to and applications of benzohydrazide derivatives, see: Bedia *et al.* (2006 ▶); Bhole & Bhusari (2009 ▶); Loncle *et al.* (2004 ▶); Raj *et al.* (2007 ▶). For the stability of the temperature controller used in the data collection, see: Cosier & Glazer, (1986 ▶).
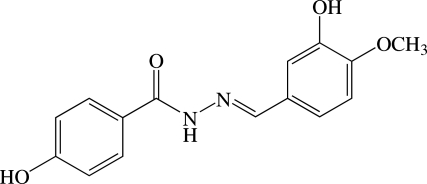


## Experimental

### 

#### Crystal data

C_15_H_14_N_2_O_4_
*M* *_r_* = 286.28
Monoclinic, 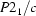

*a* = 10.7484 (5) Å
*b* = 9.4669 (4) Å
*c* = 15.7198 (5) Å
β = 122.166 (2)°
*V* = 1354.04 (10) Å^3^
*Z* = 4
Mo *K*α radiation
μ = 0.10 mm^−1^
*T* = 100 K
0.42 × 0.29 × 0.19 mm

#### Data collection

Bruker APEX DUO CCD diffractometer
Absorption correction: multi-scan (*SADABS*; Bruker, 2009 ▶) *T*_min_ = 0.958, *T*_max_ = 0.981
16525 measured reflections
3910 independent reflections
3438 reflections with *I* > 2σ(*I*)
*R*_int_ = 0.021

#### Refinement

*R*[*F* ^2^ > 2σ(*F* ^2^)] = 0.041
*wR*(*F* ^2^) = 0.125
*S* = 1.03
3910 reflections
191 parameters
H-atom parameters constrained
Δρ_max_ = 0.45 e Å^−3^
Δρ_min_ = −0.49 e Å^−3^

### 

Data collection: *APEX2* (Bruker, 2009 ▶); cell refinement: *SAINT* (Bruker, 2009 ▶); data reduction: *SAINT*; program(s) used to solve structure: *SHELXTL* (Sheldrick, 2008 ▶); program(s) used to refine structure: *SHELXTL*; molecular graphics: *SHELXTL*; software used to prepare material for publication: *SHELXTL* and *PLATON* (Spek, 2009 ▶).

## Supplementary Material

Crystal structure: contains datablock(s) global, I. DOI: 10.1107/S1600536811036579/hb6400sup1.cif

Structure factors: contains datablock(s) I. DOI: 10.1107/S1600536811036579/hb6400Isup2.hkl

Supplementary material file. DOI: 10.1107/S1600536811036579/hb6400Isup3.cml

Additional supplementary materials:  crystallographic information; 3D view; checkCIF report

## Figures and Tables

**Table 1:** Hydrogen-bond geometry (Å, °) *Cg*1 is the centroid of the C1–C6 ring.

*D*—H⋯*A*	*D*—H	H⋯*A*	*D*⋯*A*	*D*—H⋯*A*
N1—H1*A*⋯O3^i^	0.88	2.30	2.9798 (12)	134
N1—H1*A*⋯O4^i^	0.88	2.53	3.3542 (12)	156
O2—H2*A*⋯O1^ii^	0.84	1.89	2.7259 (11)	174
O3—H3*A*⋯O1^iii^	0.84	1.88	2.6762 (13)	157
C10—H10*A*⋯O2^iv^	0.95	2.58	3.4786 (14)	158
C15—H15*B*⋯*Cg*1^v^	0.98	2.85	3.7211 (16)	149

Symmetry codes: (i) 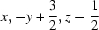; (ii) 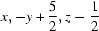; (iii) 
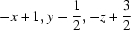
; (iv) 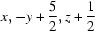; (v) 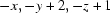.

**Fig. 1.**
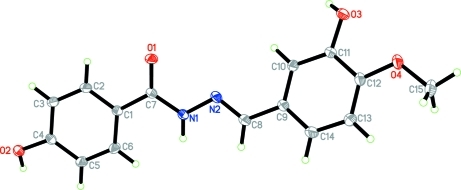
The molecular structure of the title compound, showing 50% probability displacement ellipsoids.

**Fig. 2.**
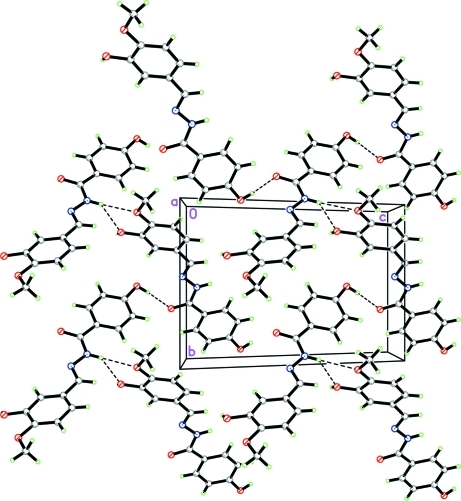
The crystal packing of the title compound viewed along the a axis, Hydrogen bonds were shown as dashed lines.

## supplementary crystallographic information

### Comment

Benzohydrazide derivatives have a wide variety of biological properties, such as antibacterial (Bhole & Bhusari, 2009), antifungal (Loncle *et al.*, 2004), antitubecular (Bedia *et al.*, 2006) and antiproliferative (Raj *et al.*, 2007) activities.

These interesting properties lead us to synthesize the title compound (I), which contains hydroxyl and methoxy substituents, in order to study and compare its biological properties with other related benzohydrazide derivatives. Herein the crystal structure of (I) is reported.

The molecule of the title benzohydrazide derivative (Fig. 1), C_15_H_14_N_2_O_4_, exists in a *trans*-configuration with respect to the C8═N2 bond [1.2811 (13) Å] and the torsion angle N1–N2–C8–C9 = 178.77 (9)°. The molecule is twisted with the dihedral angle between the two benzene rings being 24.17 (6)°. Atom O1, C7, N1, N2 and C8 of the middle bridge lie nearly on the same plane with the torsion angle O1–C7–N1–N2 = -3.15 (14)°. The mean plane through this middle bridge makes the dihedral angles of 4.82 (7) and 25.95 (7)° with the C1–C6 and C9–C14 benzene rings, respectively. The methoxy group is almost co-planar with the attached benzene ring with the torsion angle C15–O4–C12–C13 = -1.45 (17)°. Bond distances are of normal values (Allen *et al.*, 1987) and are comparable with related structures (Li & Ban, 2009; Zhang, 2011).

In the crystal packing (Fig. 2), the molecules are linked by N—H···O and O—H···O hydrogen bonds (Table 1) into sheets parallel to the *bc* plane and these sheets are further connected into three dimensional network. The crystal is stabilized N—H···O and O—H···O hydrogen bonds together with C—H···O weak interaction. C—H···π weak interaction (Table 1) was also observed.

### Experimental

The title compound (I) was prepared by dissolving 4-hydroxybenzohydrazide (0.1 mmol, 0.15 g) in ethanol (15 ml). A solution of 3-hydroxy-4-methoxybenzaldehyde (0.1 mmol, 0.15 g) in ethanol (15 ml) was then added slowly to the reaction. The mixture was refluxed for around 5 hr. The solution was then cooled to room temperature. Colorless blocks of (I) were obtained after slow evaporation of the solvent at room temperature after several days, Mp. 516 K (decompose).

### Refinement

All H atoms were positioned geometrically and allowed to ride on their parent atoms, with d(O-H) = 0.84 Å, d(N-H) = 0.88 Å, d(C-H) = 0.95 Å for aromatic and CH and 0.98 Å for CH_3_ atoms. The *U*_iso_ values were constrained to be 1.5*U*_eq_ of the carrier atom for methyl H atoms and 1.2*U*_eq_ for the remaining H atoms. A rotating group model was used for the methyl groups. The highest residual electron density peak is located at 0.70 Å from C1 and the deepest hole is located at 0.21 Å from H2A.

### Crystal data

**Table:**

C_15_H_14_N_2_O_4_	*F*(000) = 600
*M**_r_* = 286.28	*D*_x_ = 1.404 Mg m^−^^3^
Monoclinic, *P*2_1_/*c*	Melting point = 516 (decompose)–516 K
Hall symbol: -P 2ybc	Mo *K*α radiation, λ = 0.71073 Å
*a* = 10.7484 (5) Å	Cell parameters from 3910 reflections
*b* = 9.4669 (4) Å	θ = 2.2–30.0°
*c* = 15.7198 (5) Å	µ = 0.10 mm^−^^1^
β = 122.166 (2)°	*T* = 100 K
*V* = 1354.04 (10) Å^3^	Block, colorless
*Z* = 4	0.42 × 0.29 × 0.19 mm

### Data collection

**Table:**

Bruker APEX DUO CCD diffractometer	3910 independent reflections
Radiation source: sealed tube	3438 reflections with *I* > 2σ(*I*)
graphite	*R*_int_ = 0.021
φ and ω scans	θ_max_ = 30.0°, θ_min_ = 2.2°
Absorption correction: multi-scan (*SADABS*; Bruker, 2009)	*h* = −14→15
*T*_min_ = 0.958, *T*_max_ = 0.981	*k* = −13→13
16525 measured reflections	*l* = −22→22

### Refinement

**Table:**

Refinement on *F*^2^	Primary atom site location: structure-invariant direct methods
Least-squares matrix: full	Secondary atom site location: difference Fourier map
*R*[*F*^2^ > 2σ(*F*^2^)] = 0.041	Hydrogen site location: inferred from neighbouring sites
*wR*(*F*^2^) = 0.125	H-atom parameters constrained
*S* = 1.03	*w* = 1/[σ^2^(*F*_o_^2^) + (0.0719*P*)^2^ + 0.4766*P*] where *P* = (*F*_o_^2^ + 2*F*_c_^2^)/3
3910 reflections	(Δ/σ)_max_ = 0.001
191 parameters	Δρ_max_ = 0.45 e Å^−^^3^
0 restraints	Δρ_min_ = −0.49 e Å^−^^3^

### Special details

**Table:**

Experimental. The crystal was placed in the cold stream of an Oxford Cryosystems Cobra open-flow nitrogen cryostat (Cosier & Glazer, 1986) operating at 100.0 (1) K.
Geometry. All esds (except the esd in the dihedral angle between two l.s. planes) are estimated using the full covariance matrix. The cell esds are taken into account individually in the estimation of esds in distances, angles and torsion angles; correlations between esds in cell parameters are only used when they are defined by crystal symmetry. An approximate (isotropic) treatment of cell esds is used for estimating esds involving l.s. planes.
Refinement. Refinement of F^2^ against ALL reflections. The weighted R-factor wR and goodness of fit S are based on F^2^, conventional R-factors R are based on F, with F set to zero for negative F^2^. The threshold expression of F^2^ > 2sigma(F^2^) is used only for calculating R-factors(gt) etc. and is not relevant to the choice of reflections for refinement. R-factors based on F^2^ are statistically about twice as large as those based on F, and R- factors based on ALL data will be even larger.

### Fractional atomic coordinates and isotropic or equivalent isotropic displacement parameters (Å^2^)

**Table:**

	*x*	*y*	*z*	*U*_iso_*/*U*_eq_	
O1	0.43925 (8)	1.18018 (8)	0.58385 (5)	0.01852 (17)	
O2	0.49471 (9)	1.47445 (8)	0.24675 (6)	0.01967 (17)	
H2A	0.4792	1.4316	0.1952	0.030*	
O3	0.26685 (8)	0.66939 (8)	0.80751 (6)	0.01987 (17)	
H3A	0.3556	0.6774	0.8260	0.030*	
O4	0.01675 (9)	0.54612 (9)	0.72204 (6)	0.02306 (18)	
N1	0.28428 (10)	1.03431 (9)	0.45801 (6)	0.01717 (18)	
H1A	0.2380	1.0104	0.3942	0.021*	
N2	0.26221 (9)	0.96119 (9)	0.52515 (6)	0.01676 (18)	
C1	0.40755 (10)	1.22076 (10)	0.42422 (7)	0.01464 (18)	
C2	0.50952 (12)	1.33110 (12)	0.46469 (8)	0.0208 (2)	
H2B	0.5603	1.3494	0.5348	0.025*	
C3	0.53774 (12)	1.41401 (12)	0.40453 (8)	0.0229 (2)	
H3B	0.6071	1.4888	0.4334	0.027*	
C4	0.46467 (10)	1.38813 (10)	0.30166 (7)	0.01553 (19)	
C5	0.36654 (12)	1.27532 (12)	0.26061 (8)	0.0210 (2)	
H5A	0.3193	1.2547	0.1910	0.025*	
C6	0.33780 (12)	1.19320 (12)	0.32125 (8)	0.0208 (2)	
H6A	0.2699	1.1173	0.2925	0.025*	
C7	0.37956 (10)	1.14385 (10)	0.49418 (7)	0.01477 (18)	
C8	0.15580 (12)	0.87261 (12)	0.48512 (8)	0.0204 (2)	
H8A	0.0982	0.8631	0.4141	0.024*	
C9	0.12146 (11)	0.78585 (11)	0.54692 (8)	0.0189 (2)	
C10	0.21899 (10)	0.77112 (10)	0.65114 (7)	0.01542 (19)	
H10A	0.3115	0.8176	0.6838	0.019*	
C11	0.18034 (10)	0.68906 (10)	0.70614 (7)	0.01506 (19)	
C12	0.04194 (11)	0.62256 (11)	0.65889 (8)	0.0189 (2)	
C13	−0.05414 (13)	0.63664 (15)	0.55581 (9)	0.0306 (3)	
H13A	−0.1475	0.5918	0.5232	0.037*	
C14	−0.01309 (13)	0.71667 (15)	0.50042 (9)	0.0312 (3)	
H14A	−0.0781	0.7241	0.4297	0.037*	
C15	−0.12259 (13)	0.47708 (14)	0.67802 (10)	0.0297 (3)	
H15D	−0.1294	0.4289	0.7306	0.045*	
H15A	−0.1325	0.4077	0.6285	0.045*	
H15B	−0.2014	0.5473	0.6448	0.045*	

### Atomic displacement parameters (Å^2^)

**Table:**

	*U*^11^	*U*^22^	*U*^33^	*U*^12^	*U*^13^	*U*^23^
O1	0.0209 (3)	0.0232 (4)	0.0119 (3)	−0.0039 (3)	0.0090 (3)	−0.0025 (3)
O2	0.0247 (4)	0.0215 (4)	0.0148 (3)	−0.0043 (3)	0.0119 (3)	0.0004 (3)
O3	0.0180 (3)	0.0263 (4)	0.0132 (3)	−0.0006 (3)	0.0069 (3)	0.0032 (3)
O4	0.0221 (4)	0.0259 (4)	0.0225 (4)	−0.0044 (3)	0.0128 (3)	0.0058 (3)
N1	0.0221 (4)	0.0188 (4)	0.0119 (4)	−0.0044 (3)	0.0100 (3)	−0.0008 (3)
N2	0.0198 (4)	0.0186 (4)	0.0146 (4)	0.0001 (3)	0.0110 (3)	0.0020 (3)
C1	0.0162 (4)	0.0159 (4)	0.0129 (4)	−0.0002 (3)	0.0085 (3)	−0.0002 (3)
C2	0.0222 (5)	0.0248 (5)	0.0121 (4)	−0.0074 (4)	0.0069 (4)	−0.0013 (4)
C3	0.0244 (5)	0.0265 (5)	0.0142 (5)	−0.0107 (4)	0.0079 (4)	−0.0018 (4)
C4	0.0170 (4)	0.0172 (4)	0.0142 (4)	0.0007 (3)	0.0096 (3)	0.0014 (3)
C5	0.0263 (5)	0.0244 (5)	0.0131 (4)	−0.0077 (4)	0.0111 (4)	−0.0040 (4)
C6	0.0265 (5)	0.0221 (5)	0.0155 (5)	−0.0097 (4)	0.0124 (4)	−0.0055 (4)
C7	0.0158 (4)	0.0163 (4)	0.0130 (4)	0.0009 (3)	0.0081 (3)	0.0004 (3)
C8	0.0211 (5)	0.0246 (5)	0.0141 (4)	−0.0031 (4)	0.0084 (4)	0.0021 (4)
C9	0.0184 (4)	0.0219 (5)	0.0153 (4)	−0.0030 (3)	0.0083 (4)	0.0023 (3)
C10	0.0146 (4)	0.0165 (4)	0.0154 (4)	−0.0009 (3)	0.0081 (3)	−0.0003 (3)
C11	0.0155 (4)	0.0156 (4)	0.0137 (4)	0.0017 (3)	0.0076 (3)	0.0006 (3)
C12	0.0189 (4)	0.0199 (5)	0.0189 (5)	−0.0023 (3)	0.0106 (4)	0.0030 (4)
C13	0.0200 (5)	0.0428 (7)	0.0205 (5)	−0.0137 (5)	0.0050 (4)	0.0054 (5)
C14	0.0225 (5)	0.0452 (7)	0.0158 (5)	−0.0128 (5)	0.0032 (4)	0.0066 (5)
C15	0.0267 (5)	0.0295 (6)	0.0348 (6)	−0.0092 (4)	0.0177 (5)	0.0038 (5)

### Geometric parameters (Å, °)

**Table:**

O1—C7	1.2465 (12)	C4—C5	1.3946 (14)
O2—C4	1.3468 (12)	C5—C6	1.3864 (14)
O2—H2A	0.8400	C5—H5A	0.9500
O3—C11	1.3642 (12)	C6—H6A	0.9500
O3—H3A	0.8400	C8—C9	1.4616 (14)
O4—C12	1.3671 (12)	C8—H8A	0.9500
O4—C15	1.4302 (13)	C9—C14	1.3889 (15)
N1—C7	1.3518 (13)	C9—C10	1.4049 (13)
N1—N2	1.3841 (11)	C10—C11	1.3804 (13)
N1—H1A	0.8800	C10—H10A	0.9500
N2—C8	1.2811 (13)	C11—C12	1.4087 (14)
C1—C2	1.3981 (13)	C12—C13	1.3886 (15)
C1—C6	1.3992 (13)	C13—C14	1.3913 (16)
C1—C7	1.4774 (13)	C13—H13A	0.9500
C2—C3	1.3813 (14)	C14—H14A	0.9500
C2—H2B	0.9500	C15—H15D	0.9800
C3—C4	1.3927 (14)	C15—H15A	0.9800
C3—H3B	0.9500	C15—H15B	0.9800
			
C4—O2—H2A	109.5	N2—C8—C9	121.12 (9)
C11—O3—H3A	109.5	N2—C8—H8A	119.4
C12—O4—C15	116.86 (9)	C9—C8—H8A	119.4
C7—N1—N2	117.51 (8)	C14—C9—C10	119.23 (10)
C7—N1—H1A	121.2	C14—C9—C8	118.41 (9)
N2—N1—H1A	121.2	C10—C9—C8	122.36 (9)
C8—N2—N1	115.06 (8)	C11—C10—C9	119.98 (9)
C2—C1—C6	118.24 (9)	C11—C10—H10A	120.0
C2—C1—C7	116.77 (9)	C9—C10—H10A	120.0
C6—C1—C7	124.97 (9)	O3—C11—C10	124.27 (9)
C3—C2—C1	121.18 (9)	O3—C11—C12	115.25 (9)
C3—C2—H2B	119.4	C10—C11—C12	120.44 (9)
C1—C2—H2B	119.4	O4—C12—C13	125.91 (9)
C2—C3—C4	120.09 (9)	O4—C12—C11	114.55 (9)
C2—C3—H3B	120.0	C13—C12—C11	119.53 (9)
C4—C3—H3B	120.0	C12—C13—C14	119.73 (10)
O2—C4—C3	117.26 (9)	C12—C13—H13A	120.1
O2—C4—C5	123.26 (9)	C14—C13—H13A	120.1
C3—C4—C5	119.48 (9)	C9—C14—C13	121.04 (10)
C6—C5—C4	120.11 (9)	C9—C14—H14A	119.5
C6—C5—H5A	119.9	C13—C14—H14A	119.5
C4—C5—H5A	119.9	O4—C15—H15D	109.5
C5—C6—C1	120.83 (9)	O4—C15—H15A	109.5
C5—C6—H6A	119.6	H15D—C15—H15A	109.5
C1—C6—H6A	119.6	O4—C15—H15B	109.5
O1—C7—N1	120.39 (9)	H15D—C15—H15B	109.5
O1—C7—C1	121.31 (9)	H15A—C15—H15B	109.5
N1—C7—C1	118.28 (8)		
			
C7—N1—N2—C8	170.09 (9)	N2—C8—C9—C14	165.34 (12)
C6—C1—C2—C3	2.04 (16)	N2—C8—C9—C10	−14.19 (17)
C7—C1—C2—C3	−176.66 (10)	C14—C9—C10—C11	−0.25 (16)
C1—C2—C3—C4	−0.29 (18)	C8—C9—C10—C11	179.28 (10)
C2—C3—C4—O2	178.66 (10)	C9—C10—C11—O3	−179.40 (9)
C2—C3—C4—C5	−2.01 (17)	C9—C10—C11—C12	−1.60 (15)
O2—C4—C5—C6	−178.17 (10)	C15—O4—C12—C13	−1.45 (17)
C3—C4—C5—C6	2.53 (16)	C15—O4—C12—C11	179.62 (10)
C4—C5—C6—C1	−0.77 (17)	O3—C11—C12—O4	−1.13 (13)
C2—C1—C6—C5	−1.50 (16)	C10—C11—C12—O4	−179.13 (9)
C7—C1—C6—C5	177.08 (10)	O3—C11—C12—C13	179.87 (11)
N2—N1—C7—O1	−3.15 (14)	C10—C11—C12—C13	1.87 (16)
N2—N1—C7—C1	178.39 (8)	O4—C12—C13—C14	−179.17 (12)
C2—C1—C7—O1	3.16 (14)	C11—C12—C13—C14	−0.3 (2)
C6—C1—C7—O1	−175.44 (10)	C10—C9—C14—C13	1.8 (2)
C2—C1—C7—N1	−178.39 (9)	C8—C9—C14—C13	−177.71 (13)
C6—C1—C7—N1	3.01 (15)	C12—C13—C14—C9	−1.6 (2)
N1—N2—C8—C9	178.77 (9)		

### Hydrogen-bond geometry (Å, °)

**Table:**

*Cg*1 is the centroid of the C1–C6 ring.

**Table:**

*D*—H···*A*	*D*—H	H···*A*	*D*···*A*	*D*—H···*A*
N1—H1A···O3^i^	0.88	2.30	2.9798 (12)	134
N1—H1A···O4^i^	0.88	2.53	3.3542 (12)	156
O2—H2A···O1^ii^	0.84	1.89	2.7259 (11)	174
O3—H3A···O1^iii^	0.84	1.88	2.6762 (13)	157
C10—H10A···O2^iv^	0.95	2.58	3.4786 (14)	158
C15—H15B···Cg1^v^	0.98	2.85	3.7211 (16)	149

Symmetry codes: (i) *x*, −*y*+3/2, *z*−1/2; (ii) *x*, −*y*+5/2, *z*−1/2; (iii) −*x*+1, *y*−1/2, −*z*+3/2; (iv) *x*, −*y*+5/2, *z*+1/2; (v) −*x*, −*y*+2, −*z*+1.
